# Facial nerve paresis in the course of masked mastoiditis as a revelator of GPA

**DOI:** 10.1007/s00405-021-07166-w

**Published:** 2021-11-19

**Authors:** Joanna Marszał, Anna Bartochowska, Randy Yu, Małgorzata Wierzbicka

**Affiliations:** 1 Department of Otolaryngology, Head and Neck Surgery, Poznan University of Medical Sciences, 49 Przybyszewskiego Street, 60-355 Poznan, Poland; 2 Graduate of Advanced MD Program, Poznan University of Medical Sciences, Poznan, Poland

**Keywords:** GPA, Facial nerve palsy, Masked mastoiditis, c-ANCA

## Abstract

**Purpose:**

The aim of this study was to present a series of 6 patients with facial nerve palsy and masked mastoiditis which constituted as revelators of localized granulomatosis with polyangiitis (GPA) and to evaluate the utility of the ACR/EULAR 2017 provisional classification criteria for GPA in such cases.

**Methods:**

Study group included 58 patients with GPA. Cases with facial nerve palsy and masked mastoiditis were thoroughly analyzed.

**Results:**

The mean age of patients was 37 years. All manifested unilateral facial nerve palsy and hearing loss, while only 2 reported aural complaints suggesting inflammatory cause of the disease. All cases were qualified for surgical intervention. Intraoperative findings were similar: granulation tissue in tympanic cavity and/or pneumatic spaces of the mastoid process. Only 50% of histopathological results suggested vasculitis. In all cases, elevated levels of antineutrophil cytoplasmic antibodies (ANCA) against peroxidase 3 (PR3-ANCA) were determined. Two patients presented rapid progression of the disease and died within 1 week and 2 months, respectively. Four other patients manifested gradual improvement of hearing and facial nerve function after treatment.

**Conclusion:**

GPA should be included into differential diagnosis in all cases of persistent facial nerve palsy especially when otological symptoms coexist. Even localized GPA could be very aggressive, revelating generalized form of the disease. Rapid systemic treatment of GPA can protect hearing and facial nerve from permanent severe dysfunction. The ACR/EULAR 2017 provisional classification criteria for GPA seem to be valuable tool in diagnosing ENT patients with localized otological form of the disease.

## Introduction

Localized granulomatosis with polyangiitis (GPA), an autoimmune necrotizing small-vessel vasculitis, may affect ear, nose, larynx, and trachea [1]. Otologic involvement is quite common among ENT symptoms of the disease, with up to 50% of patients manifesting ear pathology [2–4]. The most common location of the otologic involvement is the middle ear––it presents as serous otitis media, chronic otitis media, or chronic Eustachian tube dysfunction [5]. The second group of symptoms concerns inner ear and manifests as sensorineural hearing loss which is very often accompanied by tinnitus or vertigo [6, 7]. Some cases of external otitis with atypical course were also reported [8]. Facial nerve palsy, usually associated with inflammation in middle ear spaces, is quite unique and can be observed in 8–10% of patients [5, 9].

Masked mastoiditis is a rare subclinical complication of acute or chronic otitis media. The oligosymptomatic nature of the complication, often accompanied by other intracranial pathologies, causes great diagnostic difficulties. In the pathogenesis of this insidious clinical entity, the aditus ad antrum is often blocked by inflammatory mucosa, granulation tissue, or cholesteatoma [10]. Symptoms caused by persistent inflammation in mastoid air cells include pain localized deep in middle ear and retroauricular region, conductive hearing loss, and recurrent fever episodes. In most patients, no otoscopic changes or only thickening of tympanic membrane is observed. This oligosymptomatic nature often delays proper diagnosis. If mastoiditis is left untreated, it can lead to other complications, including facial nerve palsy [11]. While in classic inflammatory process, the main mechanism of paresis is the direct intoxication of the nerve by pathogens [12], the process in GPA involves the inflammation and occlusion of the “vasa nervorum” segment [13]. Some authors [5, 7] still support the theory of the facial nerve damage in the mechanism of compression by granulomatous lesions, and that hypothesis probably should also be considered in the palsy which occurs in the course of mastoiditis.

Although otological manifestations are quite common in GPA, unspecific clinical picture, including facial nerve paresis, may postpone appropriate diagnosis. Delayed treatment usually leads to progression to irreversible phase of the disease. Therefore, timely diagnosis of localized form is so important.

The purpose of this study was to present a series of 6 patients with facial nerve palsy and masked mastoiditis which constituted as revelators of GPA. We also aimed to evaluate the utility of the ACR/EULAR 2017 provisional classification criteria for GPA [14] in such sophisticated cases.

## Material and methods

All patients with GPA treated in our department in 2008–2019 (58 altogether) were included into the analysis. We focused on those with facial nerve palsy and masked mastoiditis which were the first clinical symptoms of the disease. These cases were thoroughly analyzed. The following variables were collected: age, gender, symptoms, clinical picture, diagnostic parameters of GPA [level and type of antineutrophil cytoplasmic antibodies (c-ANCA)], auditory tests (subjective audiometry, tympanometry) values, result of high-resolution computed tomography (HRCT) of the temporal bone, method of treatment, intraoperative findings, histological results, and outcomes.

All data were evaluated according to 1990 criteria for the classification of Wegener’s granulomatosis (now known as GPA) established by the American College of Rheumatology (ACR) [15] (Table 1) and 2017 ACR/EULAR provisional classification criteria for GPA presented at 2016 ACR session: New Classification Criteria for ANCA-associated Vasculitis: implications for clinical practice [14] (Table 2).

**Table 1:** The ACR 1990 criteria for the classification of Wegener’s granulomatosis (now known as GPA) [15]

1) Nasal or oral inflammation
Development of painful or painless oral ulcers or purulent or bloody nasal discharge
2) Abnormal chest radiograph
Chest radiograph showing the presence of nodules, fixed infiltrates, or cavities
3) Urinary sediment
Microhematuria (> 5 red blood cells per high power field) or red cell casts in urine sediment
4) Granulomatous inflammation on biopsy
Histologic changes showing granulomatous inflammation within the wall of an artery or in the perivascular or extravascular area (artery or arteriole)
Wegener’s granulomatosis (GPA): at least 2 of 4 criteria are present Sensitivity: 88.2% and specificity: 92.0%

**Table 2:** The ACR/EULAR 2017 provisional classification criteria for GPA [14]

Item	Score
Bloody nasal discharge, ulcers, crusting or sinonasal congestion	3
Nasal polyps	− 4
Hearing loss or reduction	1
Cartilaginous involvement	2
Red or painful eyes	1
c-ANCA or PR3-ANCA	5
Eosinophil count ≥ 1 (×10^9^/L)	− 3
Nodule, mass or cavitation on chest imaging	2
Granuloma on biopsy	3
GPA: total score of at least 5 Sensitivity: 90.7% and specificity: 93.5%	

*ACR* American College of Rheumatology, *EULAR* European League Against Rheumatism, *GPA* granulomatosis with polyangiitis, *c-ANCA* cytoplasmic-antineutrophil cytoplasmic antibodies, *PR3-ANCA* ANCA against proteinase-3

This study was approved by our institutional bioethics committee.

## Results

**Fig. 1:**
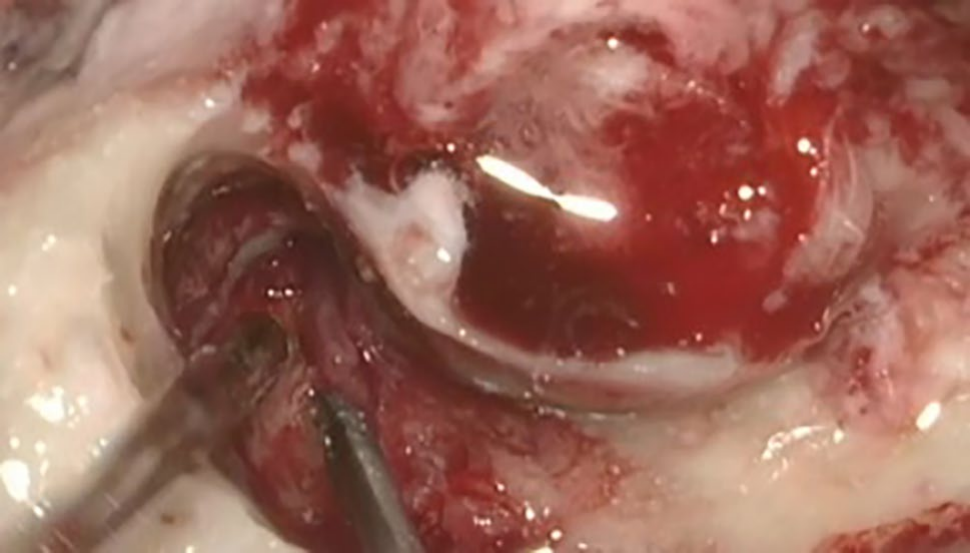
Intraoperative findings in one of our patients (No 5): granulation tissue in aditus ad antrum and in the region of facial nerve

Our study group consisted of 3 men and 3 women with a mean age of 37 years (range 31–43 years). All patients manifested unilateral facial nerve palsy and hearing loss (unilateral or bilateral, mixed type, mean hearing thresholds 75 dB, flat curves in tympanometry) lasting for 4–12 weeks. Only 2 patients reported aural complaints (discharge/pain) suggesting inflammatory cause of the disease. Otoscopic examination revealed thickening or retraction of the tympanic membrane while in one case it was reddened and another had visible scars on it with history of ear discharge. Due to the unclear clinical presentation and the radiological picture of mastoiditis in HRCT, suggesting otogenic cause of facial nerve paresis, all patients were qualified for surgical intervention. Procedures performed were the following: 3 cases––antromastoidectomy, 2 cases––canal wall up ear surgery (with partial decompression of facial nerve in one case), and 1 case––explorative tympanotomy. Intraoperative findings were similar in all patients: granulation tissue in tympanic cavity and/or pneumatic spaces of the mastoid process (Fig. 1). The lesions were partially or subtotally removed and sent for histopathologic assessment to exclude malignancy, specific, inflammatory, and autoimmunological diseases. Only 50% of results suggested vasculitis. In all patients, c-ANCA levels were evaluated: only one case had highly elevated level, four cases had slightly above normal limits, and one case had negative result at time of surgery but later turned positive after 2 months of follow-up period. In all cases, levels of ANCA against peroxidase 3 (PR3-ANCA) were determined. Although, based on ACR criteria, the diagnosis of GPA was certain only in one patient from the study group, consultant rheumatologist recommended administration of ChT (cyclophosphamide) and steroids in all c-ANCA-positive cases. In one male patient, the course of the disease was fulminant, but his general state did not allow for such therapy. Two patients presented rapid progression of the disease, and died within 1 week and 2 months, respectively. Facial nerve palsy and mastoiditis were probably the revelators of generalized form of GPA in them. Four other patients manifested gradual improvement of hearing and facial nerve function after treatment. Six months after diagnosis of the disease, the ENT examination with audiometry of these four patients revealed HBI or HBII facial nerve motoric action, and elevated pure tone audiometry curves for 20–35 dB.

Detailed clinical data are given in Table 3.

**Table 3:** The study group

	Age, gender	Symptoms	Clinical picture	Intraoperative findings	Histology	c-ANCA	Other findings	Treatment	Outcome	ACR 1990 criteria [15]	ACR/EULAR 2017 provisional classification criteria [14]
1	39 M	Bilateral hearing loss, left side facial nerve palsy (HBIII) for 2 months	Thickening of the left tympanic membrane	Tympanotomy: granulation tissue and liquid in mesotympanum, partial damage of the posterior wall of the external meatus, granulation tissue in aditus ad antrum	Not confirmed	Positive (high levels)	Elevated WBC, HRCT of the temporal bone—mastoiditis	Cht (adriamycin 90 mg iv, cyclophosphamide 1400 mg iv, and vincristine 2 mg once a day po)	Death—2 months after administration of the treatment (in the course of respiratory failure)	0/4 (diagnosis not confirmed)	6 (GPA)
2	32 M	The history of left ear pain with purulent discharge (2 months before), left facial palsy (HBVI) and hearing loss for one month	Thickening of the tympanic membrane, scars in the inferior quadrant of the tympanic membrane	Ear surgery (canal wall-up technique) with facial nerve decompression; granulation tissue in mastoid process and tympanic cavity	Confirmed	Positive	Elevated WBC, chest CT -bilateral nodules, HRCT of the temporal bone—mastoiditis	Endotracheal intubation, respiratory treatment, intravenous steroids	Death—1 week after diagnosis (in the course of respiratory failure)	2/4 (GPA)	11 (GPA)
3	42 F	Bilateral hearing loss, left facial nerve palsy (HBIII) for 6 weeks	Redness of the left tympanic membrane	Antromastoidectomy: granulation tissue in epitympanum, aditus ad antrum and antrum	Confirmed	Negative/positive in the follow-up period	HRCT of the temporal bone—mastoiditis	Oral steroids (32 mg twice a day), then cyclophosphamide	Improvement of hearing and function of the facial nerve (HBII)	1/4 (diagnosis not confirmed)	4 (diagnosis not confirmed)/9 (GPA)
4	31 M	Left facial nerve palsy (HBIII) for 3 months, unilateral hearing loss	Thickening and retraction of the tympanic membrane	Antromastoidectomy: granulation tissue in tympanic cavity and mastoid cavity	Confirmed	Positive	HRCT of the temporal bone—mastoiditis	Cyclophosphamide, prednisone	Improvement of hearing and function of the facial nerve (HBII)	1/4 (diagnosis not confirmed)	9 (GPA)
5	34 F	Right facial nerve palsy (HBIII) for one month, hypoacusis and feeling of fullness in the right ear	Retraction of the tympanic membrane	Antromastoidectomy: granulation tissue in epitympanum, mesotympanum and antrum	Not confirmed	Positive	HRCT of the temporal bone—mastoiditis	Cyclophosphamide, prednisone	Improvement of hearing and function of the facial nerve (HBI)	0/4 (diagnosis not confirmed)	6 (GPA)
6	43 F	Episode of right ear pain (1 month before), hypoacusis and facial nerve palsy (HBIV) on the right side for one month	Thickening of the tympanic membrane	Ear surgery (canal wall up technique); granulation tissue in the tympanic cavity and mastoid cavity	Not confirmed	Positive	Sinonasal congestion, HRCT of the temporal bone—mastoiditis	Cyclophosphamide, prednisone	Improvement of hearing and function of the facial nerve (HBII)	1/4 (diagnosis not confirmed)	9 (GPA)

*c-ANCA* cytoplasmic-antineutrophil cytoplasmic antibodies, *ACR* American College of Rheumatology, *EULAR* European League Against Rheumatism, *HB* *House*-*Brackmann*, *ChT* chemotherapy, *CT* computed tomography, *HRCT* high-resolution computed tomography, *GPA* granulomatosis with polyangiitis

## Discussion

The authors present a unique series of 6 patients with facial nerve palsy and masked mastoiditis which constituted revelators of GPA. Although otological complaints are quite common in GPA patients, if isolated, they can be underdiagnosed and underestimated. Facial nerve palsy, a very pronounced symptom, is very rarely related to the GPA entity.

The American College of Rheumatology (ACR) 1990 criteria for the classification of (ANCA)-associated vasculitis [15] (Table 1) seem to be not specific and sensitive enough to state the diagnosis of GPA in localized forms of the disease. In 2017, a joint working group of Diagnostic and Classification Criteria for Primary Systemic Vasculitis (DCVAS) and collaborators, including the ACR, the European League Against Rheumatism (EULAR), and vasculitis foundations, proposed the ACR/EULAR 2017 provisional classification criteria for GPA [14] (Table 2). They have not been formally published yet, but their discriminative capacity was shown to be better in many studies [16]. In our group of patients, the new classification would allow for the confirmation of GPA in all analyzed patients. Based on ACR criteria, the diagnosis was certain only in one case with fulminant course of the disease, which was in fact generalized form although otologic manifestation was initially the most expressed and pronounced. It underlines the utility of new criteria in ENT patients with otological manifestation of the disease.

The differential assessment of facial nerve palsy is complex, and very often requires many consultations and examinations before the appropriate diagnosis is determined. In unilateral involvement, 70% of cases are diagnosed as Bell’s palsy [17]. In most cases of facial nerve palsy in the course of otitis media, surgical procedure is needed. In acute infections, myringotomy is usually the first step, followed by antromastoidectomy in patients without clinical improvement. In chronic diseases, urgent mastoidectomy with the removal of granulation tissue or cholesteatoma is almost always advocated [10]. In all of our patients, middle ear was opened and granulation tissue was found along facial nerve bony canal. Only in 50% of patients, the pathological diagnosis of GPA was reached. It is in line with data presented in the literature. Most authors suggest that biopsy, which according to recommendations is the basis to establish diagnosis, may not give a definite answer in cases of localized disease. Devaney et al. [18] reported that the typical GPA picture is visible in only 25–33% of specimen taken from the middle ear. Notwithstanding, biopsy of the granulation tissue enables exclusion of malignancy and other diseases, like TBC or sarcoidosis, which makes it very important as a part of differential diagnosis scheme. A higher percentage of true positive results is noted in biopsies of the nose and paranasal sinuses. Thus, if it is possible and indicated, such a diagnostic step should also be performed [19].

Cytoplasmic pattern antineutrophil cytoplasmic antibodies are highly specific for GPA, especially in its active phase. However, they are detected in only 22–25% of patients with localized GPA manifesting as a facial nerve palsy [5]. In addition, ANCA against proteinase (PR3) and antibodies against myeloperoxidase (MPO) are detected in 80% and 10% of cases with facial nerve involvement [4]. Among the six patients, only one case presented with high levels of c-ANCA, while four had slightly elevated levels. In addition, one case was negative for c-ANCA at time of surgery, but later became positive during the follow-up period. Such a scenario was also observed by other clinicians [20]. Repeated assessment of c-ANCA is highly recommended in not-obvious, underdiagnosed cases. In all patients, PR3-ANCA were found which are also in line with literature data.

In most patients with GPA and facial nerve palsy, the paresis resolves after administration of the proper treatment [4]. Early initiation of adequate treatment tends to have better prognosis. Mur and al [8] presented a case of GPA with spontaneous improvement of facial nerve function after myringotomy and tube placement. In our patients, facial nerve palsy did not improve after surgery which prompted us for further diagnostics. Some authors underline that in active GPA, symptoms may even exacerbate after surgical procedure [21]. It is unclear whether it happens in the mechanism of “irritation” of “vasa nervorum” or progression of the disease [9]. Thus, it is highly recommended to avoid ear surgery in active disease if the diagnosis had been established. Based on the literature data and our own experience, it seems valuable to check c-ANCA levels in all patients with the clinical picture of masked mastoiditis and facial nerve palsy before surgical intervention. On the other hand, diagnostics should be expanded in all cases with atypical facial nerve palsy worsening or not improving after myringotomy or antromastoidectomy. The detailed evaluation should include MRI of the head and neck and laboratory tests to exclude viral (HIV, human herpesvirus 6, mumps virus, cytomegalovirus, and rubella virus), bacterial (Borrelia burgdorferi, *Rickettsia*, otogenic process), autoimmunological/metabolic (sarcoidosis, Sjogren’s syndrome, sclerosis multiplex, GPA), and organic (tumors in the region of cerebellopontine angle, parotid gland, petrous bone, brainstem) pathologies [22, 23]. The elevated levels of c-ANCA could indicate further diagnostic steps and enable rapid administration of the adequate treatment. Both, extended diagnostics and targeted therapy, are conducted by rheumatologists.

Nowadays, treatment of GPA involves intensive immunosuppressive therapy with high doses of corticosteroids, along with cyclophosphamide or rituximab. In severe cases, the combination of all of them is possible [24]. Once remission is achieved, patients are administered less toxic immunosuppression, such as azathioprine and low dose glucocorticosteroid [25]. Even localized disease could be very aggressive, and may lead to high morbidity [25]. It is widely discussed in the literature if toxic treatment should be administered when the diagnosis of GPA is not confirmed histologically. Many authors underline that the delay in the initiation of the treatment may negatively affect the prognosis, and thus, it should be started when c-ANCA levels are elevated and clinical picture strongly suggests GPA [19]. Truly, such cases should be diagnosed with GPA according to the new ACR/EULAR 2017 provisional classification criteria. In our group, the treatment was administered when GPA was identified in biopsy specimen or c-ANCA serologic tests were positive. Two patients presented rapid progression of the disease although the proper treatment was administered, possibly due to the cytotoxicity of the immunosuppressants, exacerbation of the disease after surgical procedure, or delayed accurate diagnosis. However, we also proved that systemic drugs allow for the improvement of hearing and function of the facial nerve in localized disease––such results were observed in all of our patients.

In the literature of the last 20 years, we have not found the descriptions of GPA cases manifesting as facial palsy and masked mastoiditis. There are several articles presenting patients with unilateral or bilateral paresis of the VIIth nerve, in most cases presenting as a sole initial symptom or as a component or complication of acute of chronic otitis media [26–28]. A wide and interesting review of such cases is presented by Iannella et al. [4]. Our study group consists of patients in whom facial nerve palsy was the first and the most pronounced symptom that brought them to the physician. Although ENT examination did not reveal emerging otoscopic changes, detailed history showed otologic origin of the paresis, and thus prompted for surgical intervention. In all cases, granulation tissue was found in middle ear spaces, and of these cases, half was identified as typical for GPA. Finally, all patients presented with positive c-ANCA. It cannot be ruled out that the prior assessment of c-ANCA could protect 5 out of 6 patients from surgical intervention. In our study group, four cases were truly localized forms of the disease and had good treatment response, which was observed as improvement of hearing and function of the VIIth nerve. In two patients, facial nerve palsy and masked mastoiditis were revelators of generalized form of GPA.

## Conclusions

GPA is an insidious and multi-symptomatic disease. It should be included into differential diagnosis in all cases of persistent facial nerve palsy especially when otological symptoms coexist. In patients with no changes in otoscopy, HRCT of temporal bone should be considered. When masked mastoiditis is suspected, c-ANCA titer should be assessed before surgical intervention. If negative, middle ear should be inspected and if granulation tissue is present, the lesion should be examined histopathologically. Even localized GPA could be very aggressive, revelating generalized form of the disease. Rapid systemic treatment of GPA can protect hearing and facial nerve from permanent severe dysfunction. The ACR/EULAR 2017 provisional classification criteria for GPA seem to be valuable tool in diagnosing ENT patients with localized otological form of the disease.

## Data Availability

Yes.

## Code availability

MS Excel.
